# Molecular vulnerabilities and therapeutic resistance in hormone receptor positive and HER2 dependent breast cancer tumours

**DOI:** 10.20517/cdr.2022.10

**Published:** 2022-06-01

**Authors:** Ravi Velaga, Sunao Tanaka, Masakazu Toi

**Affiliations:** Breast Surgery, Graduate School of Medicine, Kyoto University Hospital, Kyoto University, 54 Shogoin-Kawaracho, Sakyo-ku, Kyoto 606-8507, Japan.

**Keywords:** HER2, CDK4/6, MONALESSA-2 trial, JNK pathway, HER2 and HER3 co-mutations, microbiome, hot and cold tumour, drug resistance

## Abstract

Over the past two decades, high sensitivity to HER2-amplified primary breast cancers has been achieved with HER2-targeted therapies. CDK4/6 inhibitors have long been identified as a potential treatment option for advanced breast cancer patients. However, acquired HER2 heterogeneity leading to resistance during the treatment has been identified as a bottleneck. This review focuses on the recent resistance mechanisms identified and potential therapeutic targets for conventional and combination endocrine therapies with CDK4/6 inhibitors by various breast cancer clinical trials and research groups in HER amplified and/or mutated breast cancer tumour. Activating *HER2* alterations, JNK pathway, hyperactivated TORC1, co-mutations in *HER2* and *HER3*, phenotypic changes of HER2, and few other advanced findings are identified as potential therapeutic targets in treating current HER2 endocrine therapy-resistant tumour. Along with the HER2-focused resistance mechanisms, we also describe how the microbiome may play a role in breast cancer therapy and its potential for new therapeutic strategies to overcome drug resistance in breast cancers.

## INTRODUCTION

Human epidermal growth factor receptor 2 (*HER2*), a gene frequently implicated in breast cancers and a variety of other tumour types, results in a growth factor receptor, which, when upregulated, promotes tumour growth by inducing cell division. There has been a concerted effort to design cancer drugs that can inhibit the mutant HER2 protein, thereby successfully controlling the cell division and tumour growth. MONALESSA-2 trial^[1]^ is the first report of median overall survival exceeding five years in phase 3 postmenopausal hormone receptor positive (HR+)/human epidermal growth factor receptor 2 negative (HER2-) metastatic breast cancer (MBC) when treated with cyclin-dependent kinase (CDK) CDK4/6 inhibitor with an aromatase inhibitor. Whether this is a one-off successful trial or can be replicated in other breast cancer patients will be interesting to observe. CDK4/6 inhibitors have long been identified as a potential treatment option for advanced breast cancer patients. For nearly two decades, CDK4/6 inhibitors have become the standard of care for HR+/HER2- breast cancer. A recent study analysed the association of HER2 low expression determined by immunohistochemistry score in HR positive and HER2 negative metastatic breast cancer subjects treated with CDK4/6 inhibitors. They found that the low expression of HER2 is associated with inferior progression-free survival^[2]^. However, not all patients achieve the expected survival outcome and become resistant to treatment regimens. Further research is needed to understand how tumour cells escape the known resistance CDK4/6 mechanisms involving E2F transcriptional activity, tumour suppressor protein RB dependent cell division arrest and senescence stage, cyclin D dependent apoptosis, and others. Hence, it is important to identify novel underlying biological mechanisms leading to resistance and cancer metastasis. Some known mechanisms leading to resistance involve the role of different coregulators (e.g., AP-1, SP-1, and AIB1), kinases (e.g., *EGFR, HER2, IGF1-R, PI3K, AKT, *and* MAPK*), and loss or modification of oestrogen receptor-α (*ESR1*) while regulating the ER signalling pathway. Since *HER2* amplification alone can act as a driver in promoting carcinogenesis, targeting both HER2 activity and interacting proteins/pathways may provide a more precise and synergistic effect on tumour regression in a combination therapy setup. The use of advanced genomic technologies and computational modelling resulted in the recent reporting of potential molecular vulnerabilities and mechanisms leading to biological breakthroughs that help advance clinical trial designs and treatment of breast cancer relatively for improved response and survival rates. Many excellent reviews have covered various breast cancer molecular vulnerabilities and resistance mechanisms^[3-8]^. In this review, we focus on explaining the most recent studies that proposed resistance mechanisms in *HER2* mutated breast cancers, along with the potential use of microbiome in combinatorial immunotherapies to treat breast cancer tumour for effective response.

## HER2 DEPENDENT BREAST CANCER RESISTANCE MECHANISMS

### Activated *HER2* alterations

**Figure 1:**
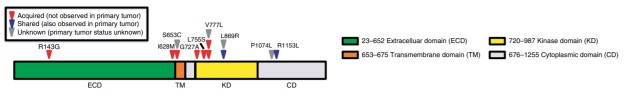
Acquired *HER2* alterations in patients with endocrine resistance. The location of *HER2* alterations identified by sequencing metastatic biopsies is depicted along the length of the protein. Protein domains are indicated by colour coding. Evolutionary classification for alterations: red triangles, acquired alterations; blue triangles, alterations shared with primary tumour; grey triangles, indeterminate or unknown. Figure 1 and legend used from Nayar *et al.*^[13]^.

Oestrogen receptor (ER), the major driver in breast cancer causation, is a known target to treat breast cancer. ER’s expression not only depends on the patient’s age and grade of the tumour but also on HER2 expression and loss of the *TP53* gene. CREATE-X study^[9]^ reported that after standard neoadjuvant chemotherapy with anthracycline, taxane, or both, the addition of adjuvant capecitabine is safe and effective in improving the disease-free survival and overall survival among patients with *HER2*- breast cancer and residual invasive disease. To understand the benefits of adjuvant therapy in primary breast cancer patients, multigene tests (e.g., OncotypeDx, MammaPrint, PAM50, and others) are being performed^[10-12]^. These multigene tests also assist in distinguishing patients who may benefit from endocrine therapy in combination with chemotherapy. These tests stratify the tumour based on the tumour grade, risk of recurrence, and likelihood of treatment response. Endocrine therapy has been shown to be effective mostly in ER+ metastatic breast cancer patients. It is known that CDK inhibitors with endocrine therapy improve outcomes in patients with metastatic ER+ breast cancer. However, its value in early-stage patients is still unclear, and research is still limited on how varying clonal events between primary and metastatic biopsies could result in developing resistance to endocrine therapies in combination with CDK inhibitors. With 25%-30% achieving response to endocrine therapy and others developing resistance, it becomes apparent that resistance to endocrine therapy develops during the treatment resulting in cancer metastasis. Beyond the historically known resistance mechanisms, few recent studies have shed light on how activating *HER2* mutations in ER+ metastatic breast cancer tumour had developed resistance to aromatase inhibitors, tamoxifen or fulvestrant^[13]^. Whole exome sequencing analysis of the primary tumour biopsies before endocrine therapy and 12 hotspot *HER2/ERBB2* mutations covering kinase, extracellular, transmembrane, and cytoplasmic domains resulted in detecting the *HER2* alterations only in the metastatic biopsies but not the primary, suggesting that these alterations were acquired during the course of therapy [Figure 1].

Clonal evolution analysis to evaluate the clonal expansion structure and dynamics also concluded similar origins of the alterations. Examination of the functional role of these alterations in T47D and MCF7 cell lines through lentiviral transduction showed strong resistance to oestrogen deprivation resistance to tamoxifen and fulvestrant. Hyperphosphorylation of both ERK and AKT under conditions of oestrogen deprivation or inhibition was also associated with the *HER2* alterations. Based on the cell viability analysis, they showed that the combined inhibition of *HER2* mutants restored sensitivity to fulvestrant. Activating *HER2* alterations are shown to offer a distinct mechanism of acquired resistance to varying ER-directed combination therapies in MBC and are postulated to be overcome by an irreversible HER2 inhibitor.

### Resistance via JNK activated pathway

**Figure 2:**
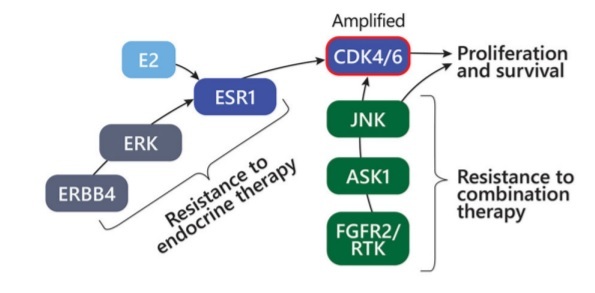
A schematic diagram showing resistance mechanisms driven by upregulation of ERBB4 and CDK6 amplification (red circle signifies amplification) or alternative signalling via FGFR2/RTK’s and JNK signal transduction. Figure used from Griffiths *et al*.^[15]^.

While the novel clonal mutations can improve understanding of the underlying resistance mechanisms and alternative therapy options, identifying the certain common characteristics or pathway alterations leading to resistance can also help offer alternative treatments. From the FELINE clinical trial^[14]^, using exome and single-cell RNA sequencing of serially collected biopsies from ER+ breast cancer patients treated with letrozole mono endocrine therapy or in combination with different doses of CDK4/6 inhibitor ribociclib, researchers tried to understand the early-stage breast cancer evolution and develop resistance. Also, the researchers examined how disruption of CDK6, Cyclin E2 and interaction of MAPK8-10, MAPK11-14, and JNK103 could result in resistance to endocrine therapy. The study^[15]^ identified JNK activation as offering an alternative mechanism to oestrogen-independent proliferation under combined therapy. Tumour cells with diminished endocrine signalling can bypass CDK4/6 inhibition through upregulation of the JNK pathway and showed that in cancer cells with high oestrogen signalling, potentiation of CDK4/6 activation can occur through ERBB4 and ERK upregulation and activation. In tumour without CDK6 amplification, the resistant cancer cell state was deemed to reflect a phenotypic shift from ERK to JNK MAPK signalling and reduced oestrogen signalling. When combination therapy was used in tumour without CDK6 amplification, JNK activation provided an alternative pathway to oestrogen-independent cell proliferation. Also, such showed less ERBB4 upregulation, indicating that JNK activation is a mechanism of resistance to endocrine but not CDK inhibition. Along with the activation of JNK signalling in tumour without CDK6 amplification with combination therapy, the absence of ERK signal further reflects the transition to an endocrine independent resistance state, with reduced reliance on the interaction of ESR1/ERK [Figure 2].

Both JNK and ERK pathways, which are major components of the MAPK network, showed differing activity patterns following combination therapy but not endocrine therapy alone. Along with the cell cycle, *TGF-β* and *TP53* pathway-related genes with frequent mutations, the majority of patients in the study maintained persistent polyclonal populations. Earlier reports demonstrated the stress kinase pathway via p38 and JNK to modulate ER function by phosphorylation of ER and its coregulators^[16-17]^. Identifying the phenotypic changes and common resistance phenotypes in the clones that survived using both exome and single-cell RNA sequencing offered an efficient method to detect resistance mechanisms. Researchers suggested that increased ERBB4 signalling, oestrogen signalling loss, responsive states, JNK pathway activation, and increase in basal-like tumour attribute as underlying features for developing endocrine therapy resistance and can be potential biomarkers while selecting and treating early-stage breast cancer patients^[15]^.

### Hyperactivated TORC1 driven acquired resistance

**Figure 3:**
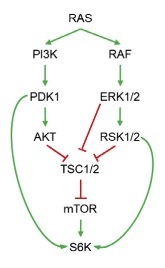
Schematic representation of RAS-mediated TORC1 activation by both PI3K and MAPK pathways. Figure used from Sudhan *et al.*^[21]^.

Neratinib, an irreversible pan-HER tyrosine kinase inhibitor, inhibits the growth of *HER2*-mutant tumour. It has been shown to be effective in preclinical studies in different tumour, including breast^[18]^, colorectal, and non-small cell lung cancers with *HER2* mutations. Following that, two clinical trials MutHER^[19]^ and SUMMIT^[20]^ explored the efficacy of neratinib in metastatic breast cancer patients with *HER2* mutations. Initial findings reported by the SUMMIT trial indicated that neratinib plus fulvestrant combination has been shown to be clinically active in heavily pre-treated *HER2*-mutant HR+ MBC patients, including in those who had received prior fulvestrant and CDK4/6i therapy with clinical benefit rate of 47% among the trial participants^[20]^. Based on the varying degrees of efficacy and clinical benefits of neratinib in *HER2* mutant cancers, Sudhan *et al*.^[21]^ postulated the role of different genomic modifiers towards the response and in developing resistance. They went on to identify clinically actionable mechanisms leading to resistance to neratinib in *HER2*-mutant cancers. Using bladder & ovarian cancer cells, they demonstrated that neratinib resistant cells showed enrichment of cell cycle promoting E2F targets, along with nuclear factor kappa B (*NF-KB*), epithelial-to-mesenchymal transition, KRAS, TORC1, inflammation, and immunological signatures. While TORC1 substrates such as p70 S6 kinase (S6K) and S6 have increased phosphorylation in drug resistance cells, EGFR, HER2, and HER3 phosphorylation were reported to be suppressed upon neratinib treatment, indicating sustained drug target inhibition. Similar activity of TORC1 was shown in neratinib-resistant breast cancer patient-derived xenografts. To confirm the role of TORC1 and to evaluate the involvement of other PI3K isoforms in neratinib’s resistance, the researchers further tested the efficacy of combining neratinib with PI3Ka (alpelisib), TORC1 (everolimus), MEK1/2 (selumetinib), AKT (MK-2206), and with the pan-PI3K inhibitor buparlisib. Pharmacological suppression testing of TORC1 resulted in identifying PI3K or MAPK inhibitors to partially suppress TORC1 activation and, hence, are less effective at reversing neratinib resistance. Further investigation revealed that both PI3K and MAPK pathways work collectively to promote TORC1 activity. Based on how RAS modulates mTOR activity through simultaneous activation of both PI3K and MAPK pathways, researchers examined RAS pathway activation status [Figure 3].

Interestingly, unlike in bladder and ovarian cancer cells, TORC1 activation in breast cancer cells was not associated with an upregulation in RAS function. This suggests that different *HER2*-mutant cancer types may develop distinct resistance mechanisms involving TORC1 signalling. The study identified hyperactivation of TORC1 as a potentially actionable mechanism driving primary and secondary resistance to neratinib in *HER2*-mutant cancers and concluded that the combination of TORC1 inhibitors with neratinib should be tested in *HER2*-mutant cancers with *de novo* or acquired mTOR pathway mutations^[21]^.

### Co-mutations in HER2 and HER3 and resistance

**Figure 4:**
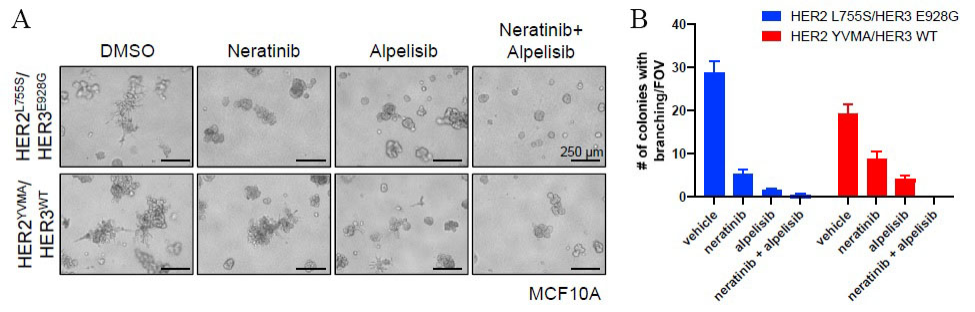
A: Shows MCF10A cells stably expressing the indicated genes were grown in 3D Matrigel in EGF/insulin-free medium + 1% charcoal/dextran-stripped serum (CSS) treated with vehicle [dimethyl sulfoxide (DMSO)], 20 nM neratinib, 1 mM alpelisib, or the combination. Scale bars, 250 µm. B: Shows the number of colonies with invasive branching per field of view (FOV) from (B) was quantified. Data represent the average ± SD (*n* = 3). Figure and legend used from Hanker *et al.*^[23]^.

SUMMIT trial^[22]^ demonstrated an effective way of probing the underlying biology among *HER2* and *HER3* mutated cancers through pharmacological HER kinase inhibition in patients. It showed an effective application of next-generation sequencing technologies in precision clinical trials while advancing our understanding of the biological impact and consequences of *HER2* and *HER3* mutations in human cancers. Although the study did not identify co-mutation of *HER2* and *HER3* in any of the study participants, concurrent aberrations in cell cycle checkpoints and activation of RTK/RAS/RAF pathway were associated with worse outcomes. While the SUMMIT trial did not report any patients with *HER2-HER3* co-mutations, another study^[23]^ reported that co-expression of mutant HER2/HER3 enhances HER2/HER3 co-immunoprecipitation and ligand-independent activation of HER2/HER3 and PI3K/AKT, resulting in enhanced growth, invasiveness, and resistance to HER2-targeted therapies. Computational modelling and *in vitro* studies of *HER2-HER3* co-mutations showed strong binding affinity and co-mutated cells grew spikes that assisted matrix invasion, a sign of metastasis [Figure 4].

The authors suggested that patients carrying *HER2-HER3* co-mutations may not be good candidates for HER2 inhibitors alone and need a new therapeutic approach in combination with TKI and PI3Ka inhibitors to suppress the HER3 activity^[23]^. Although HER3 lacks an active tyrosine kinase binding domain, it has several docking sites to bind with PI3K and form heterodimers to activate PI3K pathway and improve response to PI3K inhibitors in combination with HER2 and TKI inhibitors^[23]^.

### HER2 low positive tumour and resistance mechanisms

To further understand the use of anti-HER2 agents, Denkert *et al*.^[24]^ conducted a deep clinical and molecular analysis among 2310 patients with HER2-non-amplified primary breast cancer from four prospective, neoadjuvant trials. Based on the standardised immunohistochemistry technique, the authors reported that HER2-low-positive tumour can be identified as a new subgroup of breast cancer with distinct biology and show differences in hormone receptor status, tumour proliferation, grading, and response to neoadjuvant therapy. Significant differences in the presence of BRCA1/2 germline variants and other breast cancer predisposition genes were observed between HER2-0 and HER2-low-positive tumour. Likewise, varying frequencies of *TP53* and *PIK3CA* mutations in the two subgroups indicate different genetic and mutational backgrounds. Thus, providing enough evidence for further characterisation of the two groups to identify the mechanisms leading to sensitivity and/or resistance to treatment. A more recent study at ESMO 2021, for the first time, presented a study in which they reported a change of HER2 negative (HER2-0) to HER2 low negative tumour^[25]^. Among 547 primary breast tumour, they reported a 30% shift from HER2-0 to Her2 low expression on recurrence. This switch in HER2 expression status offers more therapeutic alternatives in HER2- and also for triple-negative breast cancer patients, in which 14% tumour reportedly had the HER2 expression change. It also shows the necessity to re-test HER2 expression at relapse for improved care.

### Beyond HER2 mutations and resistance mechanisms in hormone-receptor positive breast tumour

The past decade has shown the power of combining genomic sequencing and clinical information while elucidating the role of molecular vulnerabilities in targeted therapies. One such study analysed 1,918 advanced breast cancers, of which 692 tumour were previously treated with hormonal therapy. Using Memorial Sloan Kettering integrated mutation profiling of actionable cancer targets (MSK-IMPACT) platform, identified increased frequency of alterations in MAPK and oestrogen receptor transcriptional regulators and their exclusive occurrence with ESR1 mutations. The study identified *HER2* activating mutations, NF1 loss and EGFR amplification as potential therapeutic targets while treating the resistant tumour^[26]^. Along with understanding the role of clinically actionable mutations, it is also important to know if other variants contribute to cancer resistance. The known association of *MAP3K1, PIK3CA,* and *TP53* mutations with poor prognosis in luminal A and luminal B subtypes of breast cancer, using targeted sequencing, Griffith *et al.*^[27]^ reported the significant association of frameshift nonsense (FS/NS) mutations in *NF1* and variants of unknown significance in *PIK3R1* and *DDR1* as a marker for poor prognosis and resistance in oestrogen receptor-positive breast cancer cohort^[27-28]^. 

### Transcriptomic heterogeneity and breast cancer resistance

#### Transcriptional heterogeneity and KDM5i therapeutic resistance

Understanding the intra- and inter- tumour heterogeneity of breast tumour has helped advance our understanding of how cancers evolve and develop resistance. The role of cancer-associated genetic alterations on transcriptomes has been very well covered in earlier reviews^[29-32]^ and most recently in a study by pan-cancer analysis of a whole-genome consortium^[33]^. With growing emphasis on oncogene addiction^[34]^ and transcriptional addiction^[35]^, it becomes natural that more studies will focus on investigating various mechanisms leading to transcriptional addiction and resistance. Earlier, the generation of genome-wide maps of DNA-associated proteins to understand the dysregulated transcription and transcriptional heterogeneity was limited due to the number of cells that were needed^[35]^. However, precise measurement of transcriptional heterogeneity for effective treatment alternatives has been made possible with advances in single-cell sequencing technologies. An association of KDM5B/JARID1B, which encodes a histone H3 lysine 4 (H3K4) demethylase, with shorter disease-free survival^[36] ^was reported in breast cancer patients treated with endocrine therapy. To understand the mechanistic contribution of the KDM5 family of histone demethylases (HDMs) to tumourigenesis and therapy resistance, Hinohara *et al*.^[37]^ carried out functional studies using breast cancer cell lines. They hypothesised that modulating KDM5 activity will affect intra-cellular heterogeneity, subsequently resulting in therapeutic resistance. Using single-cell RNA sequencing and Mass spectrometry, they confirmed higher KDM5B expression levels in luminal subtypes compared with basal-like breast cancer cells. ER+ primary tumour with higher KDM5B expression levels was more likely to develop local and distant metastatic recurrence in tamoxifen-treated breast cancer patients. They also confirmed heterogeneous expression of both *KDM5B* and *KDM5A *genes. KDM5 inhibitor (KDM5i) treatment decreased cellular transcriptomic heterogeneity in luminal ER+ breast cancer cells^[37]^. Table 1 summarises the different resistance mechanisms through which breast tumour develop resistance to endocrine and combinatorial therapies. Less cellular heterogeneity could lead to more responses, thus providing evidence of an association between heterogeneity and KDM5-driven resistance.

**Table 1:** Summary of breast cancer resistance mechanisms and molecular vulnerabilities identified as potential therapeutic targets

**Resistance mechanism**	**Molecular vulnerability/potential biomarker**	**Study in…**	**Reference**
Activated *HER2* alterations	Hyperphosphorylation of both ERK and AKT	Metastatic breast cancer	[13]
Activated JNK activated	Increased ERBB4 signalling, oestrogen signalling loss, and responsive states	Early breast cancers	[14]
Hyperactivated TORC1	PI3K and MAPK pathways	*HER2*-mutant tumour	[21]
Co-mutations in *HER2* and *HER3*	Activation of HER2/HER3 and PI3K/AKT	*HER2-HER3* co-mutated tumour	[23]
HER2 low positive	Varying frequencies of *TP53* and *PIK3CA* mutations	HER2 negative tumour	[24,25]
*HER2* activating mutations, NF1 loss and EGFR amplification	*HER2* activating mutations, copy number aberrations in NF1 and EGFR	Advanced tumour with hormonal therapy	[26]
Variants of unknown significance driven resistance	Frameshift nonsense (FS/NS) mutations in *NF1, PIK3R1,* and *DDR1*	Oestrogen receptor-positive breast cancer	[27,28]
KDM5 driven resistance	Higher KDM5B expression	Breast cancer cell lines	[37]

### Dormant cells, HER2 status and resistance to endocrine therapy

Another line of thought among clinicians and researchers is whether endocrine therapy works by inhibiting cell division and/or by shifting the cancer cells to dormant cells. A study using single-cell and imaging analysis by Hong *et al.*^[38]^ investigated the contribution of clonal genetic diversity and transcriptional plasticity in both early and late phase tumour with endocrine therapy. Interestingly, they assigned therapy resistance to pre-adapted or dormant cells which had undergone transcriptomic reprogramming and copy number alterations. Earlier studies have reported that thrombospondin 1^[39]^ and integrin^[40]^ induce breast cancer cell proliferation and chemoresistance by the interaction between the cells and secreted molecules. Studies in mice have shown that some cancer cells with E-cadherin expression have activated HER2 and WNT-dependent migration^[41]^ to distant organs and progesterone-induced migration of HER2+ cancer cells resulting in distant metastasis^[42]^. This suggests that the disseminated cancer cells remain dormant for a period of adaptation and attains positive clonal selection^[43]^ before they proliferate^[44]^. The use of stage-specific pre-adapted biomarkers to characterise the pre-adapted cells could assist in detecting resistance. A recent *in vitro* study also demonstrated that activating the MET/FAK signalling axis leads to CDK4/6-independent CDK2 activation and could thus become a target to improve the response of cancers to CDK4/6-targeted therapies^[45]^.

### Tumour microenvironment, microbiome, and therapeutic resistance 

The tumour microenvironment surrounding tumour cells is extremely important for their growth. The tumour microenvironment is characterized by stroma, fibroblasts, inflammatory cells, immune cells, vascular system, and connective tissue. As each tumour is unique, the microenvironment surrounding the tumour is also extremely diverse. The interrelationship between tumour cells and the tumour microenvironment is deeply involved in tumour growth, invasion, metastasis, and antitumour drug sensitivity and resistance. Antitumour drug resistance is acquired through diverse mechanisms, such as blocking the immune clearance of tumour cells, preventing drug absorption, and stimulating paracrine growth factors to promote tumour cell growth^[46]^. Changes in the tumour microenvironment can also be attributed to the microbiome^[47]^. Microbiome can alter the tumour microenvironment by regulating circulating inflammatory and immunocompetent cells, even in a distant tumour. In treatment, the antitumour effect of cytotoxic agents is influenced by the activation of immune cells and the microbiome^[48]^. Differences in the microbiome have been found to affect antitumour efficacy. A report showed that certain types of microbiome act to enhance antitumour immunity and help immune checkpoint inhibitors. Bifidobacterium induces immune-related genes in dendritic cells and enhances the antitumour effect of immune check inhibitors by inducing the activity of CD8-positive cells^[49]^. The *Bacteroides* species, *Bacteroides thetaiotaomicron*, and *Bacteroides fragilis* enhance the effect of anti-CTLA-4 antibodies^[50]^. There is growing clinical evidence that the microbiome influences the effectiveness of tumour immunotherapy. The patients who showed drug resistance or low drug efficacy had a lower microbiome diversity^[51]^ and abundant *Ruminococcus obeum* and *Roseburia intestinalis*^[52-53]^. These studies suggest that if the antitumour immune activity is weak due to the insufficient microbiome, immune checkpoint inhibitors will not be sufficiently effective. The role of the microbiome in the efficacy of immunotherapy has been increasingly evaluated at the cellular level. Activation of macrophages and dendritic cells has been reported to be under the control of the microbiome^[54]^. The suppression of drug resistance in breast cancer could be solved by inducing the immunosuppressive “cold” tumour microenvironment, which inhibits the antitumour effect, to an immunologically active state, “hot” tumour immune microenvironment, by the microbiome. Prediction of drug efficacy and drug resistance by analysis of the intestinal microbiota and elimination of drug resistance by alteration of the intestinal microbiota may become a new therapeutic strategy in the future.

## CONCLUSION

The use of advanced tumour and single-cell technologies has resulted in redefining the HER2 expression status, thus helping in identifying more accurate treatment alternatives for increased sensitivity. Reproducing similar clinical outcomes like in MONALEESA-2 trial would be more encouraging for both clinicians and patients. For researchers, such trials provide a unique opportunity to understand and elucidate specific molecular vulnerabilities and potential therapeutic targets among non-responders and responders.

## DECLARATIONS

### Copyright

© The Author(s) 2022.
